# Processing of Red Dragon Fruit Juice by Membrane-Based Operations: A Key Factor in Obtaining Concentrated Fractions of Functional Interest

**DOI:** 10.3390/foods15101725

**Published:** 2026-05-14

**Authors:** Carmela Conidi, Alessia Ruffolo, Nguyen Van Tuyen, Chu Xuan Quang, Dang Thao Yen Linh, Alberto Figoli, Alfredo Cassano

**Affiliations:** 1 Institute on Membrane Technology “Enrico Drioli”—National Research Council of Italy (ITM-CNR), Via P. Bucci, 17C, 87036 Rende, Italy; a.ruffolo@itm.cnr.it (A.R.); a.figoli@itm.cnr.it (A.F.); 2 Center for Advanced Materials and Environmental Technology, National Center for Technological Progress, Thanh Xuan District, Hanoi 100000, Vietnam; nguyenvantuyen951994@gmail.com (N.V.T.); quangcx@gmail.com (C.X.Q.); dangthaoyenlinh@gmail.com (D.T.Y.L.)

**Keywords:** red dragon fruit juice, juice processing, ultrafiltration, osmotic distillation, betalains

## Abstract

Red dragon fruit (*Hylocereus polyrhizus*), also referred to as pitaya, is an exotic fruit rich in macro- and micro-nutrients, including powerful natural antioxidants, that brings numerous benefits to human health, mostly for the control and management of the oxidative stress. Therefore, it has a great potential for industrial exploitation aimed at maximizing the extraction of its high-value bioactive compounds, specifically betacyanins (red pigments) and phenolics, for the production of functional foods, beverages, and health products. This aim of this study was to evaluate the production of high-quality concentrated red dragon fruit juice by using an integrated membrane system based on a combination of ultrafiltration (UF) and osmotic distillation (OD) processes capable of effective, but still mild concentration of valuable juice. Specifically, after juice extraction, the raw juice was preliminarily clarified by UF and then concentrated by OD up to 41 and 50 °Brix using dehydrate calcium chloride brine as the osmotic agent. The performance of UF and OD membranes was investigated under selected operating and hydrodynamic conditions. In addition, the impact of the integrated process on the quality of clarified and concentrated juices was assessed in terms of physicochemical properties and antioxidant activity. Physicochemical parameters and antioxidant activity were largely preserved after concentration, demonstrating the effectiveness of the proposed process in maintaining the nutritional, organoleptic, and nutraceutical properties of the juice.

## 1. Introduction

Red dragon fruit (*Hylocereus polyrhizus*), also known as red pitaya, is a grapevine cactus plant fruit that grows in tropical and subtropical Countries such as Vietnam, Indonesia, Philippines, Malaysia, Thailand, Mexico, Taiwan, West Indies, and Bangladesh [1,2].

Currently, Vietnam is the main exporter of dragon fruit worldwide (more than half of global output) [3] with an average productivity of 22–35 t/ha/year [4].

The potential of red pitaya as a functional fruit has been widely recognized [5]. Specifically, its high antioxidant activity has been correlated with the presence of flavonoids and phenolic acids [6], and especially with betacyanins [7], pigments responsible for the color of this fruit and its derived products. Other important biological properties, including hypocholesterolemic, anti-inflammatory, chemopreventive, and cytotoxic activities, have also been reported [8,9,10]. The juice is an important value-added product, low in calories and cholesterol-free content, and aids as a raw material for the production of jellies, juices, syrups, and other processed products [11].

Fruit juices are widely preferred by consumers for their high bioactive potential and perceived health benefits [12]. However, a major challenge in dragon fruit processing is improving both juice yield and the recovery of bioactive compounds from the pulp. The main difficulty in juice extraction arises from the high pectin content, which is primarily composed of rhamnogalacturonans, homogalacturonans, and neutral sugars. These components form a complex polysaccharide matrix that provides structural integrity to the fruit but also increases resistance to mechanical breakage during pressing [13]. In particular, during juice extraction, pectin forms a gelatinous network that entraps water and other soluble compounds, thereby limiting their release. This gelation increases pulp viscosity and significantly hinders the separation of juice from solid residues [14]. Moreover, thermal treatments may induce degradation of the juice leading to losses in both color and antioxidant activity loss in the final products [15]. Finally, the bioactive composition of dragon fruit can be affected by several external factors, including season, weather conditions, transportation, handling, and storage [16].

In recent decades, pressure-driven membrane processes, including microfiltration (MF), ultrafiltration (UF), nanofiltration (NF) and reverse osmosis (RO), have gained significant importance in the juice industry for clarification, purification, and concentration of juices and beverages, while preserving their nutritional and sensorial compounds [17]. In particular, MF and UF membranes have been increasingly applied as alternative clarification steps to conventional flocculation with filter aids (e.g., gelatine, silica sol, bentonite and diatomaceous earth), decantation and centrifugation. These conventional operations are often limited by long operating times, significant product losses and potential health and environmental risks associated with the handling of fining agents, including dust inhalation and waste generation. MF and UF membranes enable the production of clarified juices through the removal of microorganisms, macromolecules, fibers and suspended colloids from fresh juice. At the same time, low-molecular-weight solutes such as sugars, vitamins and minerals permeate through the membrane, thereby preserving the nutritional and organoleptic properties of the original juice. In juice processing application. typical molecular weight cut-off (MWCO) range from a few kilodaltons up to pore size of approximately 0.2 μm [18].

NF and RO processes are promising alternatives to multistage thermal evaporation traditionally used for juice concentration. These processes enable shelf-life extension, reduced packaging, storage, and transport costs, and improved microbiological stability of the final products [19]. However, their application is limited by the high hydraulic pressure required for operation which limit the maximum achievable concentration. In addition, these conditions promote concentration polarization and organic fouling phenomena leading to reduced permeate flux, shortened membrane lifespan, and increased operating and capital costs [20].

Membrane contactor techniques, such as membrane distillation (MD) and osmotic distillation (OD), can significantly contribute to overcoming these limitations. These processes are driven by a vapor pressure difference across porous hydrophobic membrane surfaces through which only water vapor is transported. They operate at operating pressures and temperatures much lower than those typically applied in conventional evaporation processes, thereby effectively reducing both thermal and mechanical stresses on the treated juices [21].

To the best of our knowledge, no studies have reported the application of the OD process for the concentration of red dragon fruit juice or the corresponding quality characterization. Therefore, the aim of this study was to investigate the production of high-quality red dragon juice concentrate using an integrated membrane system as alternative to conventional processing methods, in which OD is used to concentrate the juice after a preliminary clarification of the raw juice by UF. Specifically, concentrated samples with a total soluble solid (TSS) content of 41 and 50 °Brix were produced starting from a raw juice with a TSS content of 11 °Brix, extracted from fresh fruits, preliminarily clarified by UF. The productivity of both UF and OD membranes was evaluated in selected operating and hydrodynamic conditions. The impact of both processes on physico-chemical parameters and antioxidant activity of the resulting clarified and concentrated juices was also assessed and discussed.

## 2. Materials and Methods

### 2.1. Juice Extraction

Red dragon fruits (*Hylocereus polyrhizus*) of Vietnamese origin were purchased from a local shop in Cosenza (Italy). The fruits were thoroughly rinsed with tap water, wiped dry, peeled and manually cut into pieces of suitable size. Juice extraction was performed using a centrifugal juice extractor (MMV702, Omega Juicers, London, UK) during which the juice was automatically separated from the residual pulp and seeds. To increase the extraction yield, the residual pulp was subjected to approximately 10 additional extraction cycles. The resulting mixture (approximately 15 kg from multiple extraction cycles) was incubated for 4 h at 40 °C with 20 g/kg of pectolytic enzyme (Pectinex Ultra SP-L, Novo Nordisk A/S, Novo Allè, 2880 Bagsuaerd, Denmark) enabling the hydrolysis of pectin. The hydrolyzed mixture was then pre-filtered using a 200 μm filter to remove large particles. The raw juice was stored at −20 °C and thawed to room temperature prior to use.

### 2.2. Juice Clarification

The raw juice was clarified using a laboratory-scale pilot unit equipped with a cross-flow filtration system fitted with a polyethersulphone hollow fiber membrane module (Mycrodin Nadir, FB02-FC-FUS 5082, Wiesbaden, Germany) with a MWCO of 500 KDa and a membrane surface area of 0.25 m^2^. The UF system consisted of a 25 L feed tank, a feed pressure pump, a digital flowmeter, a thermometer and two manometers for measuring inlet and outlet pressures. The feed temperature was controlled by circulating cooling water through a heat exchanger; the axial feed flowrate (Q_f_) and the transmembrane pressure (TMP) were regulated using a needle valve on the concentrate line and by setting the speed pump.

During the process the permeate was collected separately, while the retentate was continuously recycled to the feed tank in a batch concentration configuration, until a volume reduction factor (VRF) of 7 was reached. TMP, Q_f_ and operating temperature were set at 0.4 bar, 330 L/h and 26 ± 1 °C, respectively.

The hydraulic permeability of the UF membrane was determined before and after juice clarification. It was calculated as the slope of the linear relationship obtained by plotting water flux versus applied TMP. The permeability of the clean membrane was indicated as L_p_^0^, while the value measured after juice filtration was indicated as L_p_^1^. After juice clarification the UF membrane was cleaned in place following a multistep procedure: (1) an initial cleaning step with a 0.2% *w/w* NaOH solution recirculated through the membrane at 40 °C for 60 min; (2) rinsing with water at 25 °C for 10 min; (3) a second cleaning step with a 1% *w*/*w* enzymatic solution (Filzym P3, from Realzyme, Springboro, OH, USA), also recirculated for 60 min at 40 °C; and (4) a final rinsing with water at 25 °C. The hydraulic permeabilities measured after the alkaline and enzymatic cleaning were indicated as L_p_^2^ and L_p_^3^.

The fouling index (FI) indicating the decrease of the water permeability after juice clarification, was calculated using the following equation:

(1) $$FI=\left(1-\frac{L_{p}^{1}}{L_{p}^{0}}\right)\cdot 100$$

The water permeability recovery (WPR) after chemical and enzymatic cleaning was evaluated using the following equation:

(2) $$WPR=\left(\frac{L_{p}^{3}}{L_{p}^{0}}\right)\cdot 100$$

The fouling mechanism during UF of red dragon juice was also evaluated using Hermia’s model [22] based on a mathematical relationship between the cumulative permeate volume (V) and filtration time, as expressed in Equation (3):

(3) $$\frac{d^{2}t}{dV^{2}}=k{\left(\frac{dt}{dV}\right)}^{n}$$

where *k* is constant of model and the values of *n* are established for different fouling mechanisms.

Equation (3) yields four empirical models describing fouling behaviors: complete pore blocking (*n* = 2), standard pore blocking (*n* = 1.5), intermediate pore blocking (*n* = 1) and cake filtration (*n* = 0). The corresponding linearized form of these fouling models are reported below:

(4) $$\mathrm{Complete}\;\mathrm{pore}\;\mathrm{blocking}:\;Ln\left(J^{-1}\right)=Ln\left(J_{0}^{-1}\right)+kt$$

(5) $$\mathrm{Standard}\;\mathrm{pore}\;\mathrm{blocking}:\;J^{-0.5}=J_{0}^{-0.5}+kt$$

(6) $$\mathrm{Intermediate}\;\mathrm{pore}\;\mathrm{blocking}:\;J^{-1}=J_{0}^{-1}+kt$$

(7) $$\mathrm{Cake}\;\mathrm{filtration}:\;J^{-2}=J_{0}^{-2}+kt$$

where *J* and *J*_0_ are the permeate flux at *t* (h) of operating time and initial time, respectively. The experimental data from the UF process were fitted to Equations (4)–(7), and the dominant fouling mechanism responsible for flux decline was identified based on the highest correlation coefficient (*R*^2^). The mass transfer coefficients (*k*) were determined from the slopes of the corresponding linear plots.

### 2.3. Juice Concentration

The clarified juice was concentrated using a laboratory-scale OD plant consisting of two independent circuits, one for the juice and the other for the brine. Two magnetic drive gear pumps with variable speed control were used to circulate the solutions on each side of the contactor. The OD unit was equipped with a 3M Liqui-Cell Extra-Flow 2.5 × 8-in. membrane contactor (3M, Charlotte, NC, USA) containing microporous polypropylene hollow fibers with an average pore diameter of 0.2 µm and a total membrane surface area of 1.4 m^2^. The concentrate loop was continuously fed with the clarified juice at an average flow rate of 3.4 ± 0.2 L/min while a 60% *w/w* calcium chloride dihydrate (Fluka Chemie GmbH, Buchs, Switzerland) solution was used as the stripping phase and circulated counter-currently on the lumen side at an average flowrate of 1.4 ± 0.2 L/min. Both streams were recirculated to their respective reservoirs after passing through the contactor, at a temperature of 26 ± 2 °C. Inlet and outlet pressures for both tube-side and shell-side streams were monitored by pressure gauges to control the pressure differential across the membrane. Average pressures were maintained at 1 ± 0.4 bar on the shell side and 0.4 ± 0.2 bar on the lumen side.

The initial volume of the stripping solution (approximately 12 L) was two times higher compared to that of the juice, in order to prevent a significant dilution and the consequent reduction of the driving force during the process.

The water flux during concentration was determined by monitoring the decrease in juice mass over time using a balance (Gibertini Elettronica, Milan, Italy) placed beneath the juice container.

After each experimental run, both the tube and the shell sides were first rinsed with de-ionized water. Subsequently, the shell side was cleaned by circulating a 2% *w/w* KOH solution for 1 h at 40 °C, followed by a rinsing with de-ionized water and further cleaning with a 2% *w/w* citric acid solution for 1 h at 40 °C. During these steps, the tube side was continuously rinsed with deionized water. Finally, the membrane module was dried using compressed air.

### 2.4. Physico-Chemical Parameters

Feed, permeate and retentate samples were analyzed for their content of total soluble solids (TSS), suspended solids (SS), electrical conductivity (EC), pH, salinity, total color density (TCD), total carbohydrates, betalain content (Betacyanin content, Bc; Betaxanthin content, Bx), total phenolic content (TPC), total flavonoids, total anthocyanins content (TAC) and total antioxidant activity (TAA). Retentate samples obtained at 41 and 50 °Brix were diluted to 10.2 °Brix (corresponding to the soluble solids content of the clarified juice) prior to analysis, to ensure comparability among samples and to bring analyte concentrations within the linear range of the analytical methods. Dilution was performed using ultrapure water under controlled conditions, and samples were thoroughly homogenized before analysis. The dilution factor was systematically included in all calculations.

Quantitative analyses of total carbohydrates, TPC, flavonoids and TAA were performed using external calibration curves prepared from analytical-grade standards. Each calibration curve consisted of at least five concentration levels, covering the expected range of analyte concentrations. Linearity was confirmed with an R^2^ ≥ 0.995. Calibration standards were periodically analyzed to ensure instrument stability and analytical reproducibility.

All compositional analyses of fresh juice, UF and OD samples were conducted on the same biological batch obtained from the extraction procedure described above.

#### 2.4.1. Total Soluble Solids and Viscosity

TSS were quantified using refractometers with measurement ranges of 0–32 °Brix and 28–62 °Brix (Atago Co., Ltd., Tokyo, Japan). Results were expressed as °Brix, where 1 °Brix corresponds to 1 g of total soluble solids per 100 g of sample. The viscosity of OD samples was measured by using an RFS III viscometer (Rheometric Scientific, Piscataway, NJ, USA).

#### 2.4.2. Suspended Solids, pH, Electrical Conductivity and Salinity

SS were determined by centrifuging a known volume of a previously weighed sample at 6500 rpm for 20 min; the SS content, expressed as % *w/w*, was determined after removal of the supernatant. pH was measured using a SevenExcellence Multiparameter pH Meter (Mettler-Toledo S.p.A, Milano, Italia). EC and salinity were measured by using a Five Easy FE30 conductivity meter (Mettler-Toledo S.p.A., Milano, Italia).

#### 2.4.3. Total Color Density

TCD is expressed as the total absorbance values of the brown compounds, which show maximum absorbance at 420 nm and absorbance of the juice that gives its maximum at 533 nm. It was estimated by Equation (8) [23], as a function of the absorbance at 420 nm (A_420_), at 533 nm (A_533_) and at 700 nm (A_700_), as well as the dilution factor (DF):

(8) $$TCD=\left[\left(A_{420}+A_{533}\right)-2\left(A_{700}\right)\right]*F_{D}$$

#### 2.4.4. Total Carbohydrates

Total carbohydrates, expressed as g/L of glucose, were determined using the phenol–sulfuric acid method [24]. Briefly, 200 µL of sample was reacted with 200 µL of an aqueous phenol solution (5% *w*/*w*) and 1 mL of concentrated H_2_SO_4_ (95% *w*/*w*). After thorough mixing, the samples were incubated for 30 min at 30 °C, and the absorbance was then measured at 490 nm.

#### 2.4.5. Betalains

Betalains were quantified by spectrophotometric measurements (at 538 nm and 480 nm for betacyanins and betaxanthins, respectively) using a UV–Visible spectrophotometer (UV-160 A, Shimadzu Scientific Instruments, Inc., Kyoto, Japan) at 28 °C. The betalain content (BC), expressed as mg/L, was calculated using Equation (9):

(9) $$BC=\frac{A\cdot DF\cdot MW\cdot 1000}{\varepsilon \cdot L}$$

where A is the absorbance, DF is the dilution factor, MW is the molecular weight of betacyanin and indicaxanthin, with values of MW = 550 g/mol and MW = 308 g/mol, respectively, ε is the molar extinction coefficient of betanin (ε = 60,000 L/mol·cm) and indicaxanthin (ε = 48,000 L/mol·cm) and L is the path length (1 cm) of the cuvette [25].

#### 2.4.6. Total Phenolic Content

The TPC was determined using a colorimetric assay based on the oxidation of phenolic compounds with the Folin-Ciocalteu reagent and the subsequent formation of a blue phosphotungstic/phosphomolybdenum complex. The intensity of the resulting color, measured spectrophotometrically at 756 nm, is proportional to the phenolic concentration [26]. For the assay, 0.2 mL of diluted sample was mixed with 1 mL of Folin–Ciocalteu reagent (diluted 1:10 with bidistilled water), followed by the addition of 0.8 mL of 7.5% sodium carbonate solution. The mixture was vortexed for 1 min and incubated in the dark for 30 min. Absorbance was then recorded at 756 nm against a water blank. The concentration of total phenols was calculated from the standard curve obtained by using gallic acid solutions at different concentrations and results were expressed as gallic acid equivalents (mg GAE/L).

#### 2.4.7. Total Anthocyanins Content

The TAC was assessed following the procedure described by Meng et al. [27], after appropriate dilution of the samples with distilled water. It was calculated using the following equation:

(10) $$\mathrm{TAC}\;(\mathrm{mg}/100\;g)\;=\;\left[\frac{A\;\times MW\times DF}{ℇ\times l}\;\right]100$$

where *A* is the absorbance; *MW* is the molecular weight of cyanidin-3-glucose (449.2 g/mol); *DF* is the dilution factor; *Ɛ* is the molar absorptivity (26,900 L/mol^−1^); and *l* is the length of the cell (1 cm). Results were expressed as milligrams of cyanidin-3-glucoside equivalents per 100 g of sample.

#### 2.4.8. Flavonoids

The flavonoid content was determined using the colorimetric Davis method [28], with minor modifications [29]. Briefly, 0.5 mL of juice sample was mixed with 0.5 mL of 4 M NaOH and 7 mL of diethylene glycol. The mixture was incubated at room temperature for 10 min, after which the absorbance was measured at 420 nm. Quantification was performed using a calibration curve prepared with quercetin standards, and results were expressed as milligrams of quercetin equivalents per liter (mg QE/L).

#### 2.4.9. Total Antioxidant Activity

TAA was evaluated using a modified 2,2′-azino-bis-(3-ethylbenzothiazoline-6-sulfonic acid) (ABTS) radical cation decolourisation assay, in which ABTS^+^ is generated prior to reaction with antioxidants [30]. The ABTS radical cation was prepared by mixing 10 mL of ABTS stock solution with 100 mL of 70 mM potassium persulfate solution, and the mixture was incubated in the dark at room temperature for 12–16 h. Before analysis, 1 mL of the ABTS^+^ solution was diluted with phosphate-buffered saline (PBS; 9 g/L NaCl, pH 6.8, 5 mM NaH_2_PO_4_, 5 mM Na_2_HPO_4_) to a final volume of 25 mL to obtain an absorbance of 0.70 ± 0.02 at 734 nm. Subsequently, 10 mL of sample was added to 10 mL of working ABTS solution, and absorbance at 734 nm was recorded every minute for 6 min. The value measured at 5 min was used for calculations. Total antioxidant activity (TAA) was expressed as mM Trolox equivalents, using Trolox (6-hydroxy-2,5,7,8-tetramethylchroman-2-carboxylic acid) as the reference standard under identical conditions.

#### 2.4.10. Statistical Analyses

Analyses of physico-chemical parameters and antioxidant activity were performed in triplicate. Results are reported as mean ± standard deviation. One-way analysis of variance (ANOVA) was used to compare mean values, and differences were considered statistically significant at *p* < 0.05. Statistical analyses were performed with use of Microsoft Excel software (version 2604; Microsoft Corporation, Redmond, WA, USA).

## 3. Results and Discussion

### 3.1. Juice Extraction and Physico-Chemical Composition

**Figure 1:**
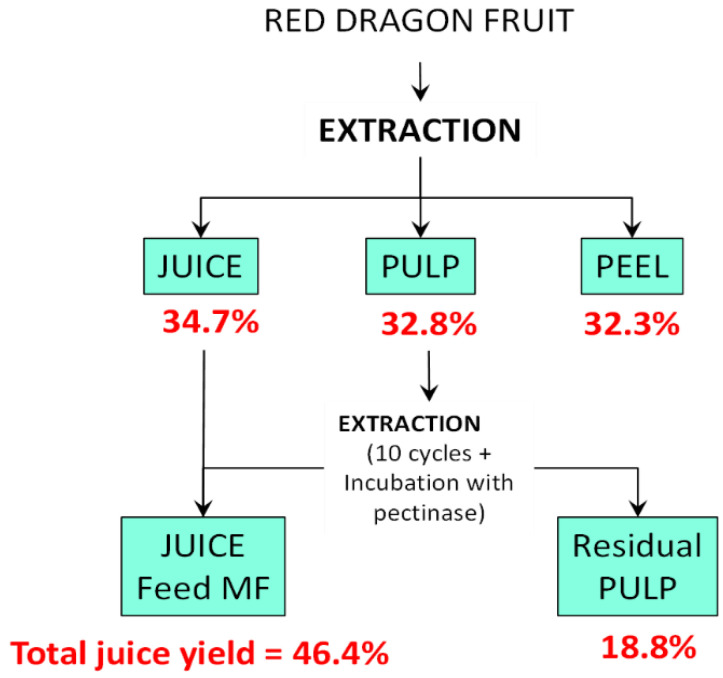
Yield (%) of the partial fractions collected from the mechanical juice extraction from red dragon fruit.

Figure 1 shows the scheme of the juice extraction process and the corresponding yields of the collected fractions. The extraction of juice from the pulp over multiple cycles (*n* = 10) increased the total juice yield from 34.7% to 46.7%. The final yield (46.7%) was significantly higher than those reported by Amorim et al. [31] for red dragon juice produced by cold pressing (16.2–20.5%) and thermo-maceration (22.3–24.1%). Previous studies also reported juice yields of 35.28% [32] with increases from 15% to 23% achieved through enzymatic cold-active extraction [33].

Table 1 summarizes the physico-chemical properties of the obtained red dragon fruit juice. The electrical conductivity (6.64 mS/cm) and salinity (3.62 psu) were slightly higher than those reported by da Costa Kassacula et al. [34]. The juice was characterized by a suspended solids content of 2.11% and a TSS content of 11.0 ± 0.01 °Brix, in agreement with data reported by Nur et al. [35] and Amorim et al. [31]. The TSS content is a key indicator of sweetness in fruits, including red dragon fruit juice.

The juice exhibited a slightly acidic pH (5.13 ± 0.03), in agreement with values reported for red dragon fruit juice [36]. Carbohydrates are one of the key macronutrients occurring in various tissues of red dragon. Together with polyphenols, they significantly influence the sensory and nutritional quality of red dragon juice [37]. The total carbohydrate content of the red dragon fruit juice extract was of 53.88 ± 2.25 g/L. Arivalagan et al. [36] reported total carbohydrate contents ranging from 5.97 ± 0.32 g/100 g FW to 6.04 ± 0.32 g/100 FW in different variety of red-fleshed dragon fruits.

Polyphenols are among the major secondary metabolites found in fruits and vegetables and are well known for their antioxidant potential. As mentioned above, dragon fruit is rich in phenolic compounds and flavonoids that exhibit various biological properties [38]. The total polyphenol content in the extracted juice was 416.87 mg/L, which is higher than that measured by Attar et al. [37] for red-purple dragon fruit juice (17.11 mg/GAE 100 g). The results reported in this study are consistent with those obtained by Abirami et al. [39], who observed polyphenol contents in the range of 32.5 and 42.5 mg/GAE/100 g. As shown in Table 1, the flavonoid content was 204.72 ± 8.75 mg/L, which is in agreement with data reported in the literature [36].

The total color density of the juice was 14.39 ± 0.34. Anthocyanins are water-soluble pigments that contribute to the coloration of fruits, including red dragon fruit juice [40]. The total anthocyanin content of red dragon juice (17.57 ± 0.17 mg cy-3-glu/100 g) was comparable to the value reported by Nur et al. [35] (16.77 ± 0.23 mg/100 g). Dragon fruit is one of the tropical fruits containing a high quantity of betalains, plant-derived pigments, increasingly used as natural colorants in the food industry. Betalains represent an important quality attribute of fruits due to their appealing coloration and their health-promoting properties [41]. Based on their chemical structure, betalains are classified into two major groups: red-violet betacyanins and yellow-orange betaxanthins [42]. In this contest, the purplish red color of dragon fruit is attributed mainly to betacyanins. As expected, the betacyanin content (103.2 ± 2.6 mg/L) was higher than that of betaxanthins (45.00 ± 0.48 mg/L), in agreement with the data reported by Zitha et al. [43].

The ABTS assay was used to evaluate the radical scavenging capacity of the red dragon fruit juice. According to several authors, the antioxidant activity of dragon fruit is attributed to the presence of both polyphenols and betalains, which can donate electrons and scavenge the ABTS radical cation [42,44,45]. The TAA of red dragon fruit juice was 2.5 mM Trolox, in agreement with values reported by García-Cruz et al. [46].

### 3.2. Juice Clarification

**Figure 2:**
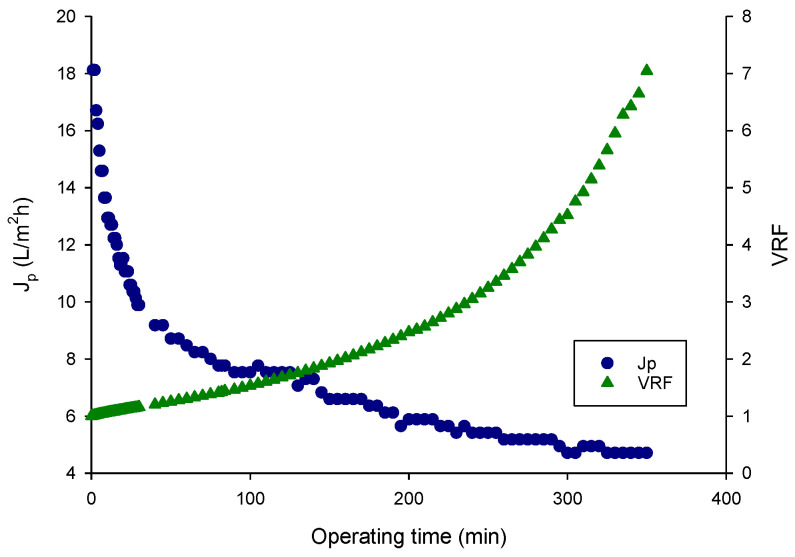
Ultrafiltration of red dragon fruit. Permeate flux (J_p_) and volume reduction factor (VRF) as a function of operating time (TMP, 0.4 bar; temperature, 26 ± 1 °C).

The raw juice was ultrafiltered in batch concentration mode under selected operating and hydrodynamic conditions. The time evolution of permeate flux and VRF is shown in Figure 2. The initial permeate flux (approximately 18 L/m^2^·h) gradually decreased over time, reaching a steady-state value of about 4.7 L/m^2^·h at a final VRF of 7. The flux decline can be attributed to a combination of effects, including concentration polarization, membrane fouling, and the increased concentration of compounds rejected by the membrane such as macromolecules, suspended solids, proteins, carbohydrates, and microorganisms [47]. These results are consistent with those reported in literature regarding the clarification of fruit juices using UF membranes. For example, similar flux declines were observed during apple juice clarification using polyethersulphone UF flat-sheet membranes (MWCO of 100, 30 and 10 kDa) [48], in cranberry juice processing with polyvinylidene difluoride (PVDF) flat-sheet UF membrane (MWCO, 50, 100 and 500 kDa) [49] and in sugar cane juice filtration using polysulphone hollow fiber membranes with MWCO values in the range of 70–100 kDa [50].

The UF membrane was cleaned according to the methodology previously described. The water permeabilities measured before and after the cleaning procedure are reported in Table 2. Specifically, the initial water permeability (*L_p_*^0^) decreased by approximately 58% after the UF process. Chemical cleaning with NaOH enabled recovery of about 73% of the initial water permeability, while the final enzymatic cleaning with Filzym P3 resulted in a finale water permeability recovery of 80%. The incomplete recovery of the initial hydraulic permeability can be attributed to irreversible fouling phenomena due to internal pore blocking and/or adsorption of foulants within the membrane structure.

The experimental data obtained during the UF process were fitted using Hermia’s fouling models. The results of fouling analysis, in terms of model fitting and correlation coefficients, are presented in Table 3. The fitting results showed that the complete pore blocking model exhibited poor correlation, with the lowest R^2^ value (0.8815), whereas the cake filtration model provided the best fit, with the highest R^2^ value (0.9882). Accordingly, cake layer formation was identified as the predominant fouling mechanism during juice clarification. This mechanism occurs when large molecules are unable to enter the membrane pores leading to the formation of a boundary layer, which in turns promotes the formation of a cake layer on the membrane surface. High-molecular-weight carbohydrates may contribute to the formation of a dense gel layer, which acts as a physical barrier and can entrap smaller, otherwise permeable phenolic molecules through physical interactions and binding [51].

The UF step can be considered a key pre-treatment for raw juice, as it enhances flow rates and maximizes process efficiency during the subsequent OD process [52].

### 3.3. Juice Concentration

**Figure 3:**
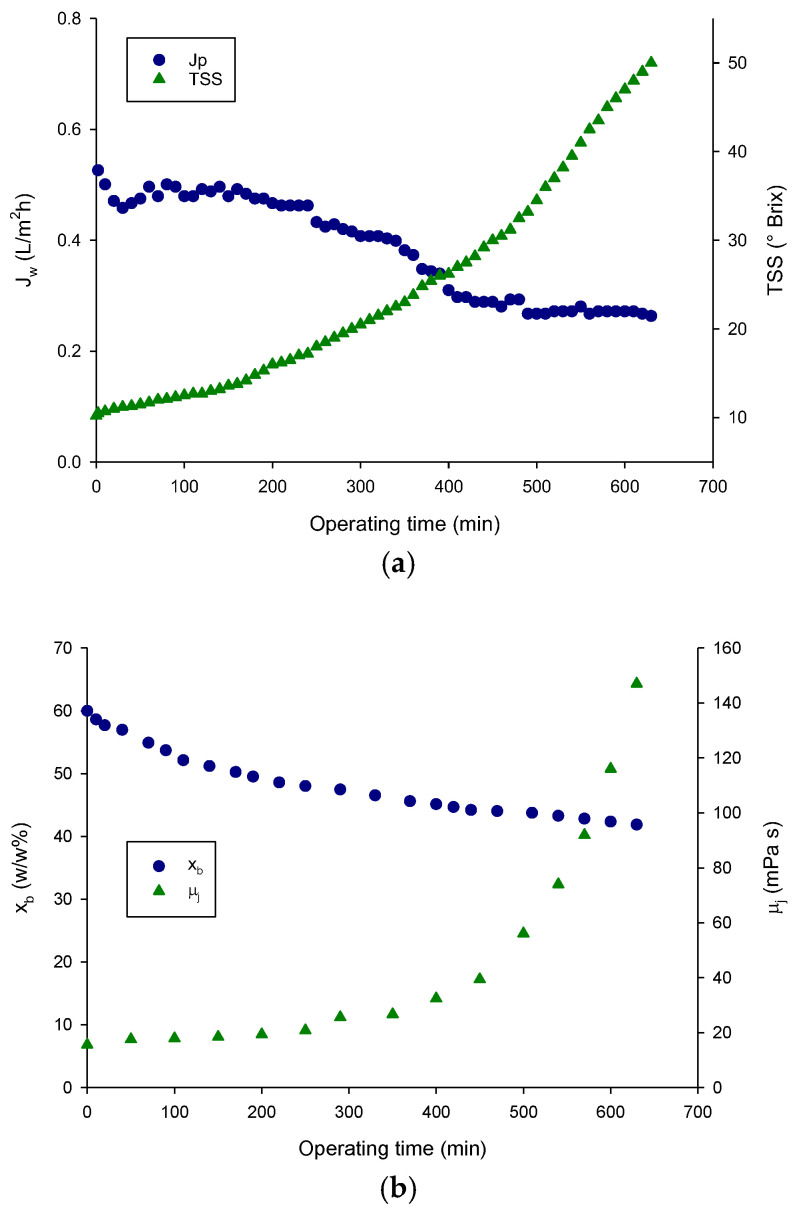
Concentration of red dragon fruit juice by OD. Time evolution of (**a**) evaporation flux (J_w_) and total soluble solids (TSS) content; (**b**) brine concentration (x_b_) and juice viscosity (μ_j_).

Figure 3a shows the time evolution of the evaporation flux and TSS content during the concentration of clarified juice up to 50 °Brix under selected operating conditions. The driving force for water transport is sustained by the difference in water activity between the juice and the brine. Specifically, water evaporates at the dilute vapour/liquid interface on the feed side, diffuses through the membrane pores and condenses at the membrane/brine interface, resulting in progressive dilution of the brine. At the end of the process the brine concentration decreased to approximately 42% *w*/*w* (Figure 3b).

The time evolution of the evaporation flux can be divided in two steps. Initially, the brine concentration was 60 *w*/*w*%, resulting in an evaporation flux of approximately 0.52 L/m^2^·h. In the time interval of 0–380 min, the decrease in evaporation flux followed a trend similar to that observed for the dilution of the stripping solution. In this range, the evaporation flux decreased by approximately 36% relative to its initial value. In the subsequent interval (380–640 min), starting from a TSS concentration of 28 °Brix, a further decline in evaporation flux was observed. This range is characterized by an exponential growth of both TSS (Figure 3a) and juice viscosity (Figure 3b), which contribute to higher resistance to mass transfer in the liquid phase. As a result, concentration polarization effects become more significant, leading to a reduction in the effective driving force. Therefore, at low TSS values evaporation flux is primarily governed by the brine concentration, whereas at higher TSS levels, flux decline is mainly influenced by increased juice viscosity and solute concentration. These findings are consistent with previous studies on OD using polypropylene hollow fiber membranes for the concentration of fruit juices such as red fruit [53], pomegranate [54], passion fruit [55] and melon [56].

### 3.4. Quality of Clarified and Concentrated Juice

Table 4 reports the values of suspended solids (SS), total soluble solids (TSS), electrical conductivity (EC) and salinity in red dragon fruit juice samples obtained after UF and OD processes. The UF process enabled the complete removal of suspended solids, producing a clear and limpid juice with a bright red color. The clarified juice exhibited a TSS of 10.2 °Brix, slightly lower than that of the raw juice. This difference can be attributed to the presence of suspended solids in the raw juice, which may interfere with refractive index measurements [57]. A slight decrease in total carbohydrate content was also observed in the UF permeate and OD samples compared to the fresh juice. The electrical conductivity and salinity of the clarified juice and concentrated samples (at 41 and 50° Brix) remained essentially unchanged. These results indicate that no significant transfer of salt compounds from the brine solution (calcium chloride dihydrate) to the juice occurred during the OD process. Furthermore, the data suggest that the hollow fiber membranes retained their hydrophobicity and structural integrity throughout the process.

A slight decrease in pH (approximately 5%) was observed in the clarified juice, likely due to the partial retention of phenolic compounds, as discussed below. In contrast, the pH values of both OD retentate samples were comparable to that of the fresh juice.

Table 5 reports the total color density (TCD) and betalain content (betacyanin and betaxanthin) in clarified and concentrated samples. A reduction of less than 20% in total betalains (16.8% for betacyanin and 19.2% for betaxanthin) was observed in the UF permeate compared to fresh dragon fruit juice. Although betalains have molecular weights lower than the pore size of the UF membrane, the observed decrease can be attributed to their interactions with compounds rejected by the membrane (i.e., carbohydrates). Similarly, a reduction of approximately 30% in betacyanin content was reported during the clarification of red-fleshed dragon fruit juice using a 10 kDa membrane [15]. Compared to clarified juice, the betacyanin content in OD samples at 41 and 50 °Brix decreased by 11% and 14%, respectively, while betaxanthin levels were slightly higher in both concentrated samples. Nevertheless, the betacyanin-to-betaxanthin ratio in these fractions remained constant. These results are consistent with previous studies reporting the stability of betalains during concentration of clarified cactus pear and red fruit juices by OD [53,58]. In contrast, significant betalain degradation has been reported during thermal processing. For example, a reduction of approximately 59% in betacyanins was observed during thermal pasteurization of red dragon fruit juice at 85 °C for 30 min [59]. Trishitman et al. [60] reported a 40% decrease in total betalain content in beetroot juice concentrated from 5 °Brix to 57.6 °Brix by thermal evaporation. Betalains are heat-sensitive pigments, highly susceptible to oxidation; their stability decreases at elevated temperatures and their degradation rate increases with prolonged heating [61].

Regarding color measurements, a decrease of approximately 17% was observed during the concentration process by OD. This behaviour could be attributed to the reduction in betacyanin content, which, as previously reported, is the major pigment responsible for the red coloration of dragon fruit. A reduction of color and betacyanin content (approximately 33%) has been reported during thermal treatment (90 °C for 60 min) of red-flesh dragon fruit purees [62].

A strong correlation between changes in betacyanin content and color in dragon fruit juice has also been demonstrated in previous studies [63,64].

Table 6 shows the total polyphenol content, total anthocyanin content (TAC) and total flavonoid content in samples obtained after UF and OD processes. Although the clarified juice showed a reduction in polyphenols (approximately 18%), these compounds were very well preserved during the OD process. Indeed, their concentrations in juice samples concentrated at 41 °Brix and 50 °Brix, were comparable to those observed in the clarified juice. In contrast, Chen et al. [65] reported a decrease in polyphenols during both traditional thermal processing (pasteurization) and non-thermal processing (ultrasonication) of red dragon fruit juice.

The UF membrane retained approximately 11% of the flavonoids present in the fresh juice. These compounds were well preserved in the concentrated fractions obtained by OD, regardless of the final concentration level. In contrast, a reduction of approximately 40% in flavonoids was reported during the concentration of red dragon fruit juice by pasteurization, when the temperature increased from 66 °C to 90 °C and the processing time from 5 to 15 min [66]. A partial retention of anthocyanins (approximately 26%) by the UF membrane was also observed. However, the subsequent OD did not significantly affect their content, which remained comparable to that measured in the clarified juice. Similar findings have been reported for cranberry juice concentrated by OD (from 8.6 °Brix to 48 °Brix): the anthocyanin content was effectively preserved and was not significantly influenced by the process [67].

**Figure 4:**
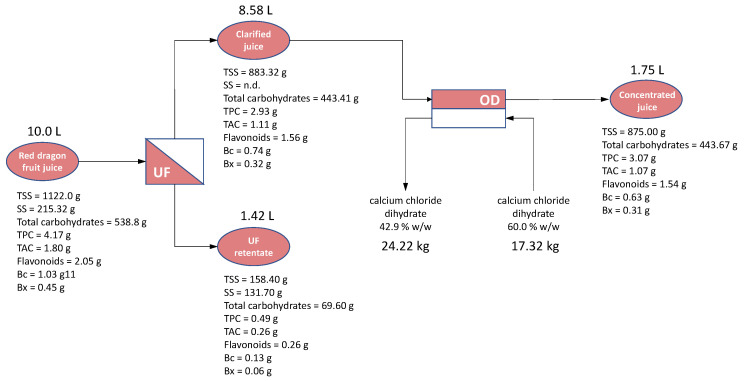
Mass balance of integrated UF/OD process for the production of concentrated juice at 50 °Brix (TSS, total soluble solids; SS, suspended solids; TPC, total phenol content; TAC, total anthocyanins content; Bc, betacyanins; Bx, betaxanthins).

The mass balance of the integrated process, estimated for the treatment of 10 L of fresh juice, ultrafiltered up to a VRF 7 and then concentrated up to 50 °Brix, is presented in Figure 4. The mass balance indicates that the recovery of bioactive compounds in the clarified juice exceeded 70% for most components, except for TAC, for which a recovery of 61% was observed. The incomplete recovery of these compounds can be attributed to their interactions with fouling agents retained by the UF membrane, as well as with membrane material itself. Carbohydrates, suspended solids, and pectin residues are known to form fouling layers on the membrane surface due to their large molecular size and gelling properties. These findings are consistent with the results of the Hermia’s model, which identified cake layer formation as the predominant fouling mechanism during UF treatment. In contrast, based on the mass balance results, the content of all analysed compounds in the concentrated juice at 50 °Brix was well preserved compared to that of the clarified juice.

**Figure 5:**
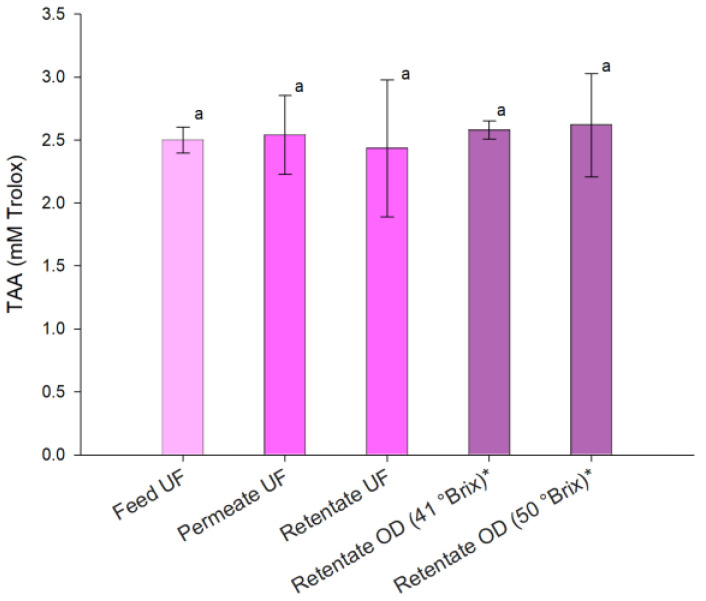
Antioxidant activity in samples of red dragon juice processed by UF and OD. * Samples diluted at 10.2 °Brix. Data are reported as the mean ± standard deviation (*n* = 3). Means with a common letter ‘a’ indicate no statistically significant differences at *p* < 0.05.

Temperature has been widely reported as a key factor affecting the stability of anthocyanins. Thermal processing promotes degradation pathways such as deglycosylation, polymerization, and molecular cleavage, resulting in anthocyanin loss and, in the presence of oxygen, browning reactions. These processes result in a reduction of color intensity and pigment concentration [68,69]. Figure 5 presents the total antioxidant activity (TAA) in the samples obtained during the UF/OD process.

The TAA of the clarified juice, closely associated with its betalain and polyphenol content, remained comparable to that of the raw juice, indicating that the UF process effectively preserved their antioxidant potential. The concentrated samples at 41 °Brix and 50 °Brix retained the TAA of the clarified juice, highlighting the mild nature of the OD process and its ability to maintain biologically active compounds. In contrast, a decrease in antioxidant activity with increasing temperature and exposure time was reported during thermal concentration (70–90 °C, 1–5 min) of dragon fruit-based beverages [11]. Similarly, Liaotrakoon et al. [70] observed a significant reduction in the antioxidant properties of dragon fruit purée during thermal treatment at 70 °C for 60 min. As antioxidative compounds are heat-sensitive, thermal processing is one of the most important factors affecting the antioxidant potential of fruit juices.

### 3.5. Sustainable Membrane-Based Production of Functional Red-Dragon Fruit Concentrates

The growing global demand for functional foods and beverages has intensified scientific and industrial interest in ingredients enriched in bioactive compounds that provide health benefits while meeting consumer preferences for plant-based and environmentally sustainable products. In this context, functional beverages represent the largest, most dynamic, and fastest-growing segment of the functional food sector. Their expansion is primarily driven by technological and logistical advantages, including packaging versatility (various sizes, shapes, and material formats), ease of storage and distribution under both refrigerated and ambient shelf-stable conditions, and high formulation flexibility [42]. Moreover, functional beverages provide an efficient delivery matrix for the incorporation of bioactive compounds and micronutrients, frequently enhancing their dispersion, stability and bioavailability. In this regard, red dragon fruit has emerged as a promising raw material for the development of functional beverages, nutraceutical formulations, and natural colorants, due to its high content of betalains, particularly betacyanins, polyphenols (mainly anthocyanins and flavonoids) and dietary fiber. Among these compounds, betacyanins can be exploited in functional food formulations, contributing to food security strategies aimed at preventing non-communicable diseases associated with oxidative stress, in alignment with the objectives of the United Nations 2030 Agenda for Sustainable Development [71].

Several studies have investigated the properties of red dragon fruit betacyanins as natural red colorants and bioactive compounds, highlighting their role in enhancing the functional properties of food matrices, particularly through increased antioxidant activity and their potential as multifunctional ingredients [72,73].

**Figure 6:**
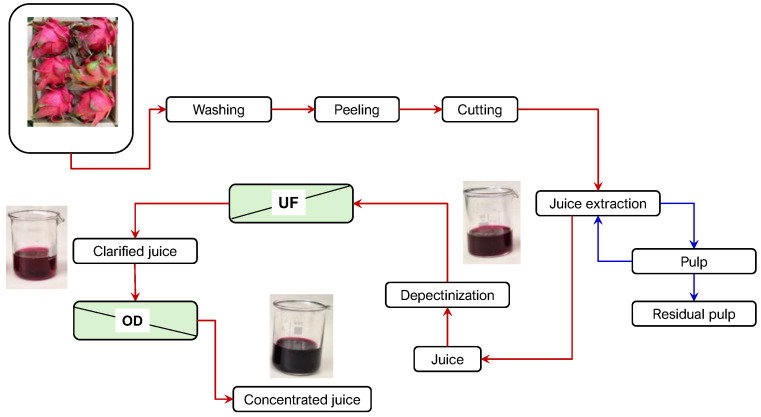
Schematic representation of the integrated UF-OD process for the production of bioactive-rich red dragon fruit concentrates.

However, despite the relatively high betacyanin content of red dragon fruit compared to many other natural sources, the absolute concentration may still be insufficient to achieve optimal health-promoting effects without appropriated formulation strategies. Moreover, the stability of these pigments is affected by several factors, including high temperatures, acidic pH, light exposure, oxygen, and high-water activity. These conditions can induce degradation reactions leading to color loss, reduced antioxidant activity, and decreased bioactive potential, thereby limiting the functional efficacy and commercial applicability of betacyanin-rich ingredients in food and beverage systems [74,75]. To address these limitations, the development of red dragon fruit-based functional beverages using non-thermal or mild processing technologies is of considerable interest. Membrane-based processes offer significant advantages in preserving thermolabile bioactive compounds while improving industrial feasibility [76]. Based on the obtained results, Figure 6 illustrates the membrane-based process designed for the production of high-quality concentrated red dragon fruit juices. The UF step produces a clarified permeate that is microbiologically stable and retains flavor, betalains, and other bioactive compounds, while microorganisms and suspended solids are retained in the UF retentate. This approach minimizes thermal exposure of the juice, thereby reducing the degradation of biologically active compounds and preserving both functional and sensory quality. The subsequent OD process produced a syrup-like juice concentrate with enhanced pigment retention, reduced water activity, and extended shelf life. Owing to its high betalain content, the concentrate can be used as a natural food colorant, offering strong coloring efficiency, an attractive hue, and good stability, thereby representing a viable alternative to synthetic colorants. From a nutraceutical perspective, it also provides bioactive compounds with potential antioxidant, hepatoprotective, and other health-promoting effects. These characteristics highlight the potential of red dragon fruit concentrate as a versatile, multifunctional ingredient for the development of next-generation functional foods, beverages, and nutraceutical formulations. Moreover, the integrated UF/OD membrane process aligns with global sustainability goals by reducing thermal energy consumption, decreasing transportation costs through volume reduction, and enabling the efficient utilization of dragon fruit resources. With further optimization to enhance membrane performance, the integrated UF/OD process offers a scalable and environmentally sustainable platform for the production of high-value functional ingredients within the rapidly expanding functional food sector.

## 4. Conclusions

The findings of this study highlight the advantages of combining ultrafiltration and osmotic distillation for the production of concentrated fractions of functional interest from red dragon fruit juice. Specifically, concentrated samples with a TSS content of 41 and 50 °Brix were obtained by recirculating the ultrafiltered juice in the shell side of the OD membrane module, while calcium chloride dehydrate solution was used as the stripping phase in the lumen side.

The OD concentration did not significantly affect the levels of phenolic compounds—particularly flavonoids—or betacyanins, regardless of the final concentration achieved, confirming the mild nature of the treatment. Consequently, the antioxidant activity of the clarified juice was largely retained in the concentrated product.

Overall, the proposed approach represents a promising alternative for preserving valuable bioactive compounds in red dragon fruit, particularly betacyanins and phenolics, thereby contributing to the efficient exploitation of this fruit for the development of functional foods, beverages, and health products. Relatively low evaporation fluxes, compared with reverse osmosis and thermal evaporation, still limit the industrial implementation of OD. In addition, the management of the spent osmotic agents or their reinforcement during OD processes remains an important research topic to reduce energy consumption and enable large-scale applications. Hybrid process configurations, involving juice preconcentration by nanofiltration or reverse osmosis followed by further concentration via OD, may represent a promising strategy to reduce processing costs and improve overall process efficiency.

## Figures and Tables

**Table 1:** Physico-chemical composition of red dragon fruit juice (TSS, Total soluble solids; EC, electrical conductivity; TPC, total phenolic content; TCD, Total color density; TAC, Total anthocyanin content; Bc, Betacyanins; Bx, Betaxanthins; TAA, total antioxidant activity). Data are reported as the mean ± standard deviation (*n* = 3).

Parameters	Value
SS (%)	2.11 ± 0.01
TSS (°Brix)	11.0 ± 0.2
EC (mS/cm)	6.64 ± 0.06
pH	5.13 ± 0.03
Salinity (psu)	3.62 ± 0.12
Total carbohydrates (g/L)	53.88 ± 2.25
TPC (mg GAE/L)	416.87 ± 17.83
Total flavonoids (mg QE/L)	204.72 ± 8.75
TCD	14.39 ± 0.34
TAC (mg cy-3-glu/100 g)	17.57 ± 0.17
Bc (mg/L)	103.2 ± 2.6
Bx (mg/L)	45.0 ± 0.5
TAA (mM Trolox)	2.5 ± 0.1

**Table 2:** Water permeability, fouling index and water permeability recovery of UF membrane.

Water Permeability (L/m^2^hbar)				FI (%)	WPR^a^ (%)	WPR^e^ (%)
L_p_^0^	L_p_^1^	L_p_^2^	L_p_^3^			
536.1	225.72	396.48	430.12	58.0	73.0	80.0

L_p_^0^ initial water permeability; L_p_^1^ water permeability after juice clarification; L_p_^2^ water permeability after alkaline cleaning; L_p_^3^ water permeability after enzymatic cleaning; FI, fouling index; WPR^a^, water permeability recovery after alkaline cleaning; WPR^e^, water permeability recovery after enzymatic cleaning.

**Table 3:** Measures of fit to the experimental data obtained for Hermia’s model: modelling of flux and values of R^2^. Experimental conditions: TMP, 0.4 bar; Q_f_, 330 L/h; T, 26 ± 1 °C.

Fouling Mechanism	Modelling	R^2^
Complete pore blocking *n* = 2	ln(*J*^−1^) = −2.4993 + 0.1897 *t*	0.8815
Standard pore blocking *n* = 1.5	*J*^−0.5^ = 0.2851 + 0.0337 *t*	0.9297
Intermediate pore blocking *n* = 1.5	*J*^−1^ = 0.0795 + 0.0245 *t*	0.9629
Cake layer formation *n* = 0	*J*^−2^ = 0.005 + 0.0068 *t*	0.9882

**Table 4:** Analysis of pH, Total Soluble solids (TSS), total carbohydrates, suspended solids (SS), electrical conductivity (EC) and salinity in samples of red dragon juice processed by UF/OD process.

Sample	pH	TSS (°Brix)	Total Carbohydrates (g/L)	SS (% *w*/*w*)	EC (mS/cm)	Salinity (psu)
Feed UF	5.13 ± 0.03 ^a^	11.0 ± 0.2 ^a^	53.88 ± 2.25 ^b^	2.11 ± 0.10	6.64 ± 0.08 ^ab^	3.62 ± 0.03 ^ab^
Permeate UF	4.87 ± 0.04 ^b^	10.2 ± 0.2 ^b^	51.68 ± 2.79 ^ab^	n.d.	6.82 ± 0.10 ^a^	3.73 ± 0.08 ^a^
Retentate UF	5.43 ± 0.01 ^c^	11.0 ± 0.2 ^a^	49.01 ± 1.80 ^a^	9.15 ± 0.45	6.43 ± 0.13 ^b^	3.50 ± 0.10 ^b^
Retentate OD (1) *	5.18 ± 0.03 ^a^	41.0 ± 0.2 ^c^	48.19 ± 1.24 ^a^	n.d.	6.90 ± 0.08 ^a^	3.75 ± 0.07 ^a^
Retentate OD (2) *	5.23 ± 0.03 ^a^	50.0 ± 0.2 ^d^	51.74 ± 1.92 ^ab^	n.d.	6.70 ± 0.13 ^ab^	3.62 ± 0.05 ^ab^

* Samples diluted at 10.2 °Brix. n.d.: not detectable. Data are reported as the mean ± standard deviation (*n* = 3). Values within a column with different letters are significantly different at *p* < 0.05.

**Table 5:** Analyses of Total Color Density (TCD) and Betalains (Betacyanin, Bc; Betaxanthin Bx) in samples of red dragon juice processed by UF and OD.

Sample	TCD	Bc (mg/L)	Bx (mg/L)
Feed UF	14.39 ± 0.34 ^a^	103.18 ± 2.59 ^a^	45.00 ± 0.48 ^a^
Permeate UF	12.58 ± 1.48 ^b^	85.68 ± 3.68 ^b^	36.37 ± 2.94 ^b^
Retentate UF	13.29 ± 0.15 ^c^	95.06 ± 0.27 ^c^	45.10 ± 1.17 ^a^
Retentate OD (1) *	10.90 ± 0.87 ^d^	75.93 ± 2.58 ^d^	38.11 ± 1.28 ^b^
Retentate OD (2) *	10.09 ± 0.30 ^d^	73.70 ± 0.64 ^d^	37.13 ± 0.70 ^b^

* Samples diluted at 10.2 °Brix. Data are reported as the mean ± standard deviation (*n* = 3). Values within a column with different letters are significantly different at *p* < 0.05.

**Table 6:** Analysis of Total phenolic content (TPC), Total anthocyanins content (TAC) and Flavonoids in samples of red dragon juice processed by UF and OD.

Sample	TPC (mg GAE/L)	TAC (mg cy-3-glu/100 g)	Flavonoids (mg QE/L)
Feed UF	416.87 ± 17.83 ^a^	17.57 ± 0.17 ^a^	204.72 ± 8.75 ^a^
Permeate UF	341.96 ± 1.36 ^b^	12.84 ± 0.26 ^b^	181.66 ± 9.82 ^b^
Retentate UF	348.63 ± 2.23 ^c^	18.44 ± 0.41 ^c^	183.88 ± 34.65 ^ab^
Retentate OD (1) *	370.98 ± 17.99 ^c^	12.40 ± 0.08 ^b^	184.44 ± 9.66 ^b^
Retentate OD (2) *	358.43 ± 21.76 ^bc^	12.51 ± 0.29 ^b^	180.28 ± 32.62 ^ab^

* Samples diluted at 10.2 °Brix. Data are reported as the mean ± standard deviation (*n* = 3). Values within a column with different letters are significantly different at *p* < 0.05.

## Data Availability

The original contributions presented in this study are included in the article. Further inquiries can be directed to the corresponding authors.

## Abbreviations

The following abbreviations are used in this manuscript:

Bc	Betacyanins
Bx	Betaxanthins
EC	Electrical conductivity
FI	Fouling Index
MWCO	Molecular weight cut-off
OD	Osmotic distillation
SS	Suspended solids
TAA	Total Antioxidant Activity
TAC	Total Anthocyanins Content
TCD	Total Color Density
TMP	Transmembrane pressure
TPC	Total Phenolic Content
TSS	Total Soluble Solids
UF	Ultrafiltration
VRF	Volume Reduction Factor
